# Brand Knowledge and Organizational Loyalty as Antecedents of Employee-Based Brand Equity: Mediating Role of Organizational Culture

**DOI:** 10.3389/fpsyg.2022.872871

**Published:** 2022-06-13

**Authors:** Xiaoming Liu

**Affiliations:** School of Marxism, Shandong Academy of Art, Jinan, China

**Keywords:** employee-based brand equity, organizational loyalty, brand knowledge, organizational culture, hospitality sector

## Abstract

This study tends to explore the impact of brand knowledge and organizational loyalty under the mediating role of organizational culture on employee-based brand equity (EBBE). For this purpose, employees of the hospitality sector were contacted to collect data through personally administrated questionnaires. Already established scales were used to devise instruments. Data were collected in two waves to minimize the common method bias. In the first wave, a total of 600 questionnaires were distributed, out of which 400 were received back, while in the second wave, remaining respondents were approached who have filled the survey in the first wave, and only 320 were received back, from which the partial and incomplete questionnaires were discarded, and at the end, 306 questionnaires were left. These final and completed responses were used for the data analysis and inferential purpose in this study. Collected data have been analyzed through Structural Equation Modeling by using Smart PLS 3 software. The assessment of measurement and structural model indicated a good model fit, and results indicate that EBBE is influenced by organizational loyalty and brand knowledge positively. Moreover, the mediating role of organizational culture has also been proved.

## Introduction

Brand equity and the value given to a product are the major indicators of commercial success and are considered as the company's most valuable resource (Boukis and Christodoulides, 2020), while the literature has suggested that a value may be added by a variety of stakeholder groups, i.e., the bulk of published scientific studies and brand equity from customer or firm's perspective (Christodoulides and de Chernatony, 2010; Veloutsou and Guzman, 2017). Workers' importance in fulfilling the marketing goals to different customers (such as clients) is extensively established, especially in the context of services. Staff skills and expertise, for instance, have a crucial impact on consumers' brand experiences and overall brand perceptions (Wallace et al., 2013). However, insufficient research has been conducted on how to improve employee-based brand equity (EBBE) so employees may effectively execute their duty as endorsers (Baker et al., 2014; Helm et al., 2016; Morokane et al., 2016).

Brands are made up of a combination of practical and emotional characteristics. To put it another way, a brand blends practical and emotional values to make a commitment well about brand image. As a result, a brand's success is determined by how well it delivers on its promises. For hospitality, however, delivering value propositions is more difficult. For starters, hospitality services are often performances or experiences that are difficult to evaluate due to their intangible character. As a result, communicating brand benefits to consumers is more complicated (Erkmen, 2018; Dinçer et al., 2020). Furthermore, because consumption and production are inextricably linked, personnel who provide services are frequently mistaken for the service itself (Erkmen, 2018). Finally, uniformity in hospitality services is difficult to attain because service delivery is dependent on personnel performance.

It is well-understood that the contact between staff and customers is critical to the consistent supply of both emotional and functional values. As a result, branding hospitality services takes a different strategy than branding physical commodities. Because hospitality service firms rely significantly on their workers to fulfill the marketing goals, there is a growing worry about how to manage their brand-related behavioral patterns (Andaji Garmaroudi et al., 2021; Buhalis and Park, 2021; Robinot et al., 2021; Wu et al., 2021). Resultantly, internal branding has already been brought to the branding literature, which is concerned with matching staff activities with brand promise (Kaushal and Srivastava, 2021; Solakis et al., 2022). Companies may utilize brands as a tool to gain more customers. The value of brands and the need for investment in brands and customers have been acknowledged by business leaders (Lim and Brown-Devlin, 2021; Martillo Jeremías and Polo Peña, 2021).

Consumer-based brand equity is becoming more of a concern, but the role of workers is also becoming more prominent (Christodoulides and de Chernatony, 2010). Employees' capacity to execute client expectations is the foundation for building a strong brand and delivering perceived service excellence (King et al., 2013). As the focus of attention changes more and more to employees, experts argue that studying brand equity from the standpoint of employees, dubbed EBBE, is vital (Gounaris, 2006; Mo et al., 2021). Enhancing EBBE helps organizations recruit competent individuals, and workers' skills and experience provide them with a competitive edge. Professionals' identification with enterprises, in contrast, may add to client satisfaction since they connect directly with consumers or customers (Poulis and Wisker, 2016).

The EBBE, as defined by King, is “the unequal influence of brand knowledge on an employee's responsiveness to internal brand management.” Moreover, how employees become related to brand values remains a key subject of study. As a result, the notion of brand equity has grown in prominence as a prerequisite for effective internal brand management. In contrast, two widely accepted approaches on brand equity continue to dominate literary works: customer-based brand equity (CBBE) and financial-based brand equity. That is why King and Grace came up with the idea for the third point of view. The authors advocated EBBE in their groundbreaking study, which emphasizes brand expertise as the cornerstone to inside brand-building initiatives (King et al., 2013; Erkmen, 2018). Given the notion's inception and the trend toward that third perspective for brand equity, most research, at present, has focused on the idea theoretically or conceptually (King and Grace, 2005, 2008, 2009, 2010; King et al., 2012). Based on this gap, this research focused on identifying the role of brand knowledge on EBBE. Human resource management is becoming a competitive edge in today's business environment.

Unsuitable employees can lead to business failure. Normal employees can keep the firm running and get it to the top, but exceptional employees may take even moderate enterprises to the top. To be successful in the future, corporations must not only hire the finest people but also make them loyal to the business to maintain them (Vardarlier, 2016; Alshraideh et al., 2017; Uzair et al., 2017). Three variables commonly determine organizational loyalty: affiliation with and conviction in the company's aims, values, and goals; proclivity to engage in activities that are profitable first and foremost to the organization; and proclivity to stay and work in the organization (Alshraideh et al., 2017). Walton was one of the first scholars to recognize the importance of loyalty (Walton and Limited, 2006). According to him, an effective performance increases when it shifts from a traditional influence strategy to a loyalty-based approach in employee management (Sokro et al., 2021; Hosseini, 2022).

Rightfully, the level of commitment is a set of techniques to analyze employee behavior and approaches with distinct gaps in the outcomes when used. To put it succinctly, organizational loyalty has a direct impact on occupational efficiency, resulting in lower employee turnover, more efficient resource use, and increased productivity (Armstrong and Kepler, 2018; Reus et al., 2019). The organizational culture component, which evolved from Hofstede's study in the 1970's and 1980's, is based on the conception of culture (Hofstede, 1980). It has become a contentious and significant topic in management study and practice. Organizational culture represents the shared values, conventions, and assumptions inside an organization, according to the concept of culture (Bharadwaj, 2014). Seemingly, organizational culture is ingrained in cultural identity, and in some cases, such as transnational corporations, it may be ingrained in many national cultures. These beliefs, attitudes, and actions of organizational personnel assist them to comprehend how the organization works are referred to as organizational culture (Schneider et al., 2012, 2017; Bataineh et al., 2017).

Organizational climate has been studied before in the context of organizations' performance, but it could have played a mediating role suggested by Schneider et al. (2017) in developing EBBE. Therefore, the author utilized it as a mediator for identifying the connecting link between brand knowledge, organizational loyalty, and EBBE. This research was based on several questions: What could be the antecedents of EBBE in the hospitality sector? How these factors, such as brand knowledge and organizational loyalty, could lead to EBBE? and What driving role, organizational culture could play between antecedents and EBBE? To address these questions, this study explored the relationships of brand knowledge, organizational loyalty, and EBBE. This study also contributed to identify the shaping role of organizational culture among the relationships of brand knowledge, organizational loyalty, and EBBE.

## Theoretical Framework and Literature Review

Brand knowledge is being used to develop overall brand recognition to the allocation of public consumption patterns, and it is also utilized to put workers' brand-related job behaviors in jeopardy. Similarly, EBBE refers to the employees' identification with the brand. In the literature, there are two viewpoints to describe the employer–employee relationship: social-exchange-based and organizational-identification-premised connections. The social exchange theory (SET), which describes workplace relationships *via* the trade of physical resources, has evolved into a social-exchange-oriented approach (Ashforth and Mael, 1989). In contrast, identification-based relationships are founded here on social identity theory (SIT), which explains employee relationships as a match among personal and corporate identities (Erkmen, 2018). SIT is the foundation of this research. The SIT was used in a variety of settings, including the psychology of consumers, information dissemination, and the connection between sports franchises and their supporters (Dimofte et al., 2014; Mckinley et al., 2014; Ambrose and Schnitzlein, 2017).

The SIT has also been used as the major conceptual framework in the study of a few hospitality researchers. SIT is a core theory in cognitive science that has been used to explain group psychology, interacting, and social perspectives, and it was proposed by Tajfel and Turner (2004). The component of one's self-concept that stems from social groups or groups to which someone belongs, as well as the significance and psychological value linked with affiliation to an organization, is referred to as social identity. It is the aspect of self-identity that is mostly generated from belonging to a group (Tajfel and Turner, 2004). People tend to associate and link themselves to diverse social groups as a way of selecting self-identity and a feeling of belonging, according to the SIT. The personal self has been founded on the importance and significance that employee puts on group identity, according to the theory. As a result, humans form a sense of social identity regarding the social characteristics of the groups to which they belong, such as race, ethnicity, gender, and political party (Chan, 2016).

The concept of social identity is crucial because it aids in understanding how inhabitants' culture and social identities impact their experiences and attitudes (Sharpley, 2014). The SIT emphasizes that a person's sense of belonging to a particular group motivates them to participate in a marketing context. This is relevant to this research, which shows the impact of organizational culture on EBBE in a hospitality solution provider. It is a concept that may be utilized to describe the themes that motivated employees must be committed to a certain brand. As a result, knowing this idea might be beneficial to hospitality sector professionals (González-Rodríguez et al., 2019; Kaur et al., 2020; Kumar and Kumar, 2020; Wang et al., 2021). Furthermore, comprehending the SIT's arguments improves one's capacity to assess the mediating impact of organizational culture in affecting EBBE. Based on the significant roles of SET and SIT in shaping EBBE, the following research evaluated the association of brand knowledge and EBBE along with the mediating role of organizational culture.

### Association of Brand Knowledge With EBBE

With the advent of an outside to the inside business standpoint, service businesses have begun to regard customer support representatives as their internal clients. As a result, employee perceptions of brand equity, a form of consistent branding activities, have begun to gain traction (Yang et al., 2015). The notion is described as “the distinctive influence that brand awareness has on an employee's reaction to internal brand management,” according to King, who saw the need for a third strategy (Keller, 1993). King and Grace claimed that staff brand equity is driven by brand knowledge, predicated on the notion of CBBE. Such that, King and Grace's method is founded on the notion that great brand equity is really the outcome of brand knowledge impacts, such as job characteristics and brand recognition (King et al., 2012). Thus, according to the study by Keller (2003), brand knowledge includes “all descriptive and evaluative brand-related information, as well as the personal meaning about a brand retained in consumer memory.”

Even though the term refers to customers, the notion is also applicable to workers because brand awareness is the cornerstone for building brand equity. Similarly, employees who are familiar with the brand are more likely to grasp their responsibilities and execute on the brand promise (Mangold and Miles, 2007; Erkmen, 2018). As a result, brand expertise combined with precise comprehension aids employees in overcoming ambiguity and committing to the brand (de Chernatony and Segal-Horn, 2001; Kumar and Kaushik, 2020; Liu et al., 2020; Osei-Frimpong et al., 2020). In terms of confusion, supplying workers with enough information regarding brand expectations will improve overall role clarity or reduce their role ambiguity. More precisely, contextual performance is a method for companies to evaluate the impact of brand awareness. Employees who are well-informed on the brand's values and expectations are more likely to absorb them and pass them on to clients (Business et al., 2018; Clark et al., 2020).

Furthermore, employees who seem to be knowledgeable and confident in their positions are more likely to form a bond with the firm and its brand. Considering the significance of attachment development, a few academics coined the term “employee brand commitment,” which they described as “the level of workers' psychological attachment to the brand, which determines their readiness to go above and beyond to achieve the brand's goals.” Scholars defined brand commitment as “the degree to which workers identify and are associated with their brand experience, are willing to expend extra efforts to achieve the brand's objectives, and are inclined to stick with the service organization” in accordance with this description (Kimpakorn and Tocquer, 2009; Reis et al., 2021). Based on these definitions, dedication is definitely the most critical consideration in determining equity. As previously mentioned, providing brand awareness allows employees to have a clear understanding of brand standards. Employees would acquire a sense of loyalty to the brand if the knowledge communicated about the branding is appreciated (Erkmen, 2018). The following hypothesis was suggested in regard to examining the relationship between brand knowledge and EBBE in the hospitality sector.

***H***_**1.**_ *Brand knowledge has a positive association with employee-based brand equity*.

### Association of Brand Loyalty With EBBE

For academics and researchers alike, organizational loyalty remains a fascinating issue. Whether from the standpoint of an employee or a consumer, loyalty is a vital measure of a successful partnership (Bahri-Ammari et al., 2016; Lim, 2016). Employee retention is a long-term goal for corporate management, whereas customer retention is a long-term goal for employees who provide high-quality hospitality. Loveman (1998) argued that when management uses internal branding methods to recruit new workers or internal branding tactics to keep good employees, loyalty is created and assessed by employee pledges and service duration. Employees are motivated to become a member of a specific brand *via* group conversations, training, seminars, and other tactics that assist them to operate according to brand promises (Loveman, 1998; Book et al., 2019). Employee loyalty *via* equity, according to Echchakoui, arises when an employee demonstrates loyalty to a firm, which improves the outcome of all attempts, such as training, role clarification, and briefing, that an organization has used on its personnel (Echchakoui, 2015).

Few researchers described that loyalty is the most essential element of the ideal company culture and that a loyal employee stays a valuable workforce (Echchakoui, 2015). Employee loyalty toward brands is increased when firms focus on connecting human resources with the company and is to deliver *via* internal branding (Du Preez et al., 2017). Emotions are linked to humans, according to the notion of emotional contagion, and hence, staff loyalty is conveyed to consumers as well. Similarly, Bitner (1990) demonstrated that branding and marketing efforts affected customer purchasing decisions and long-term connections with businesses, which stemmed from staff loyalty and dedication to their jobs. Tschirhart et al. (2005) maintained that devoted personnel perform more effectively and that this devotion not only delivers a greater return on investment for a firm but also leaves clients with a positive and emotional impression (Stock et al., 2016). Based on the significance of organizational loyalty toward strengthening the brand, the author proposed the following hypothesis.

***H***_**2.**_ *Organizational loyalty has a positive association with employee-based brand equity*.

### Mediating Role of Organizational Culture

Culture is an all-pervading phenomenon in human experience that has proven difficult to describe and quantify. Culture can be described as a determinant as well as a result of social identity. This identity also serves as a connection between someone's self-portrait and the architecture and intellectual dynamics of the social groupings. It is thought that an individual will develop a social identity through referencing to, adhering to, and emphasizing aspects of resemblance with other people. Basic premises or preconceptions, conventions, and beliefs among groups, which Hofstede defines as culture, are examples of these points of similarity (Hofstede, 1980; Fellows and Liu, 2013; Smaldino, 2019). Researchers acknowledge the importance of identity, arguing that people's judgments and actions are generally impacted by and consistent with social identities.

The organizational culture component, which evolved from Hofstede's study in the 1970's and 1980's, is based on the conception of culture (Hofstede, 1980). It has now become a contentious and significant topic in managerial study and practice. Organizational culture represents the shared values, conventions, and assumptions inside a company, according to the concept of culture (Fellows and Liu, 2013; Smaldino, 2019). Seemingly, organizational culture is ingrained in cultural identity and particular subcultures. International organizations, for example, might be embedded in far more than just national culture. These beliefs, opinions, and actions of organizational personnel assist them to comprehend how the organization works are referred to as organizational culture (Theurer et al., 2018). Each business has its own “environment,” which implies that knowing the organizational culture is critical for any company looking to achieve a competitive advantage through product innovativeness (Xie et al., 2019).

As a result, organizations with good cultural support are better positioned to achieve positive results in terms of employee behavior, perspective, ethical conduct, morality, and job satisfaction, positioning them for increased long-term corporate competitiveness and success (Wahyuningsih et al., 2019). Organizational culture, as per researchers, has two emphases, one outside as well as the other internal. The outward focus is represented by the flexibility and mission aspects, while the internal focus is represented by the involvement and consistency dimensions. Adaptability and engagement together characterize the organization's degree of flexibility, while the mission and consistency aspects combined show an emphasis on stability (Denison et al., 2012). A few researchers (Alsheikh et al., 2018; Saleem and Ilkhanizadeh, 2021) have identified the mediating role of organizational culture in different perspectives, but no one has evaluated the mediating role of organizational culture between brand knowledge, organizational loyalty, and EBBE; therefore, the author devised the following hypotheses for evaluating the aiding role of organizational culture.

***H***_**3.**_ *Organizational culture mediates the relationship between brand knowledge and employee-based brand equity*.
***H***_**4.**_ *Organizational culture mediates the relationship between organizational loyalty and employee-based brand equity*.

**Figure 1:**
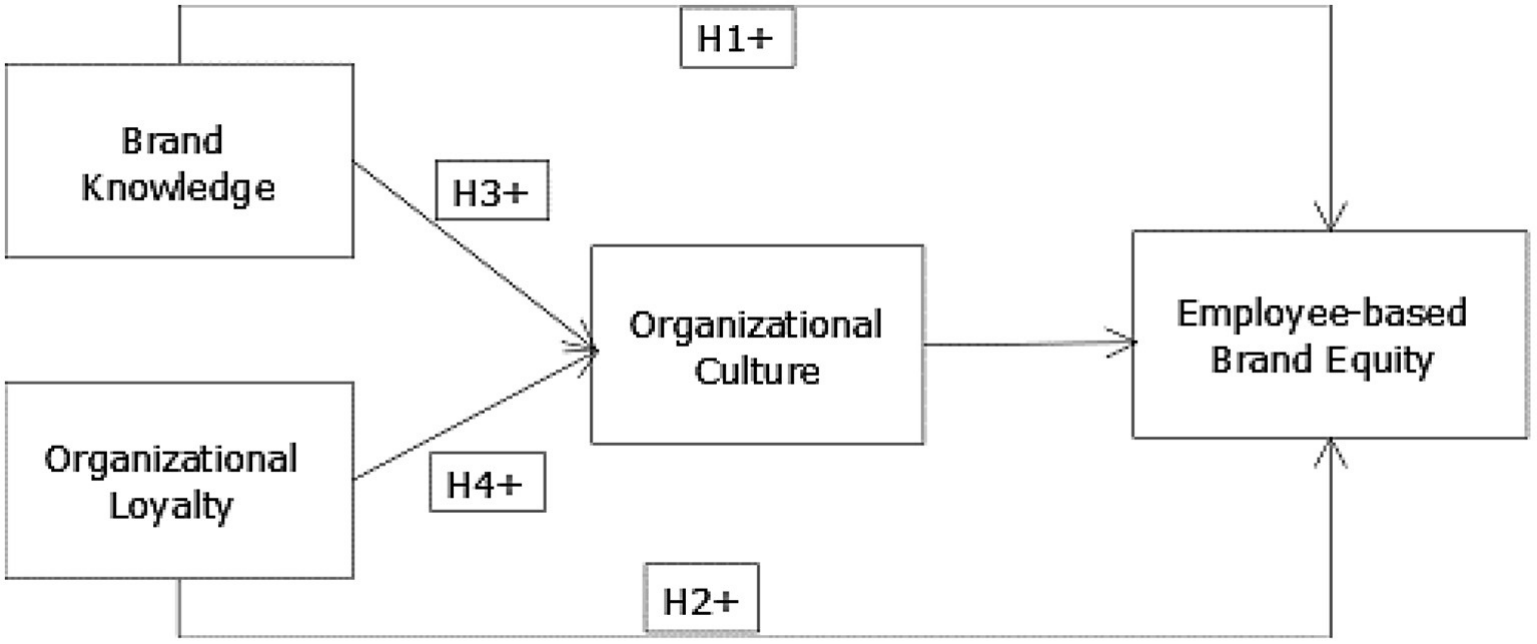
Conceptual framework.

A following conceptual model (Figure 1) has been formed based on the abovementioned literature and hypothesis.

## Methodology

### Participants and Procedure

Participants of this study were contacted based on personal contacts, and in this regard, employees of the hospitality sector were approached. Data were collected in two waves to reduce the issue of common method bias. Initially, formal approval was obtained from the concerned restaurant manager, and later on, employees were contacted, they were first briefed about the nature and purpose of the study, and their informed consent in this regard was obtained. In the first wave, data related to the independent variables of this study (brand knowledge and organizational loyalty) were obtained from the respondents. Initially, 600 questionnaires were distributed in the first wave, out of which 400 were received back. A secret identification code was allotted to each questionnaire so that the respondents could be traced easily later on. In the second wave, the remaining respondents were approached who had filled the survey in the first wave. At this time from the distributed 400 questionnaires, only 320 were received back, from which the partial and incomplete questionnaires were discarded, and at the end, 306 questionnaires were left.

These final and completed responses were used for the data analysis and inferential purpose in this study. This sample size represents the required response rate sufficiently because, according to the general rule of thumb, there are required 5–10 responses for each study variable. In this study, there are totally four constructs, and thus, a sample size of 50 would have been sufficient. Similarly, the author has employed the partial least square (PLS) approach through Smart PLS, which can handle the small sample size very comfortably. Additionally, the other criteria were also met in this regard for selecting a suitable sample size (Krejcie and Morgan, 1970). Respondents in this study were frontline workers, and it was perceived that they might be busy in their jobs due to exposure with the customers at the front line. Thus, it was the chance that and might possibly they try to fill the questionnaire through monotonic responses. Thus, the issue of common method bias possibly would have shattered the results, so this study used reverse-coded questions to minimize the monotonic responses in the data (Malhotra et al., 2006; Ng and Feldman, 2013).

#### Demographic Details

The demographic characteristics of the respondents were also obtained. Initially, the gender of the respondent was asked from the respondents. In addition to this, age and experience in years were also enquired from the respondents. From the perspective of gender, 77% of respondents were male while 23% of respondents were female. The experience was enquired in two dimensions: (1) their total experience and (2) experience in the current firm. It was operationalized that data will be collected only from those respondents who have experience of more than 3 years in the current organization so that they could provide better information regarding organizational loyalty and EBBE.

### Measures

Data in this study were obtained from the respondents on a 5-point Likert scale, and already established and tested scales have been adopted in this study to operationalize the study constructs. In this regard, the first exogenous construct of this study, i.e., brand knowledge, was measured based on the 4-item scale (Aurand et al., 2005). Previously, other scales measuring brand associations have been used in the literature, and the author has taken only dimensions related to brand knowledge and covering internal branding (Esch et al., 2006). Similarly, the second exogenous construct of this study, i.e., organizational loyalty, has been measured based on the scale developed by Van Dyne et al. (1994) and recently used by Jauhari and Singh (2013). This scale has six items that cover the concept of organizational loyalty (see Appendix).

The mediating variable of this study (organizational culture) has been measured based on two dimensions, namely, trust and respect for the individuals. Originally, this scale was developed by Ghosh and Srivastava (2014) and it covers seven dimensions of organizational culture, while the author has conceptualized only those two dimensions that were best fitted in the context of this study. Sample items include “Most people in my organization can be relied upon to keep their promises,” and “I believe that my colleagues are well-intentioned individuals.” Finally, the outcome variable of this study, i.e., EBBE, is measured based on the 5-item scale developed by Baumgarth and Schmidt (2010). A sample item for this scale includes “I am aware that everything I say or do can affect the brand image”.

## Data Analysis and Results

Keeping in view the complexity of the conceptual framework, the author has employed a multivariate data analysis tool based on the PLS approach. The most common and frequently used tool in this regard is Smart PLS, and the author has used this statistical software (Smart PLS 3.9) to assess the model (Sarstedt et al., 2014). One more reason in this regard was based on the data normality issue, as Smart PLS deals very well with the non-normal data, and the issue of normality does not influence the predictive capability of the model (Hair et al., 2017). Moreover, one other reason was based on the theoretical contribution of the study, because theory in case of EBBE is less developed, and thus, using the PLS approach provides benefits in this regard. PLS approach assesses the model in two dimensions: (1) assessment of the measurement model and (2) assessment of the structural model. The measurement model is assessed to confirm the quality criteria, based on reliability and validity (Hair et al., 2017).

**Figure 2:**
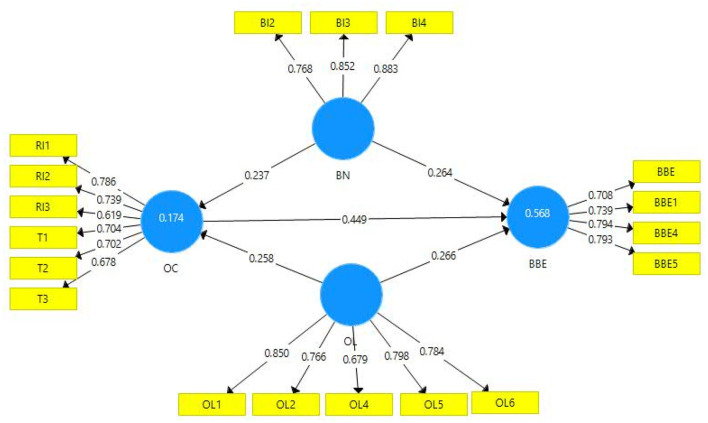
Path diagram.

To assess the measurement model, the statistics related to reliability and validity were checked, and it was found that all the indicators pertaining to the reliability and validity measures were intact (Table 1). Reliability statistics in terms of alpha range from 0.764 to 0.836. A higher level of reliability was observed for construct organizational loyalty while a lower level was observed in case of the brand-based equity. Similarly, the second measure of reliability analysis was also depicting a reasonable level of reliability (ranging from 0.774 to 0.843). The third measure of reliability statistics, i.e., composite reliability, was also depicting a good and reasonable level of 0.845–0.884. Thus, all the measures of reliability have indicated a satisfactory and sound level of reliability, while in case of validity, the first measure of validity was checked based on average variance extracted (AVE). In this regard, the AVE of all the study constructs was within the acceptable range (>0.50). Thus, a reasonable and sufficient level of convergent validity was explained (Table 1), and more than 50% of the variance in the study constructs was shared. The second measure of convergent validity relates to the outer loadings. For the assessment of convergent validity based on outer loadings, items with poor loadings were traced and were dropped from further analysis. One item from the construct brand knowledge was dropped (BI-1) due to weak and poor outer loadings (loadings <0.708) (Mela and Kopalle, 2002). Similarly, the second construct of this study organizational loyalty was checked for poor outer loadings, and item OL-3 was dropped due to less and poor loadings. One item from the study constructs EBBE was dropped (BBE-2) from the analysis due to poor loadings. No item was dropped from the study construct organizational culture (covered through dimensions, trust, and respect for the individuals; Table 2 and Figure 2).

**Table 1:** Reliability and validity.

**Construct**	**Cronbach's alpha**	**rho_A**	**Composite reliability**	**Average variance extracted (AVE)**
Brand-based equity	0.764	0.774	0.845	0.577
Brand knowledge	0.787	0.830	0.874	0.698
Organizational culture	0.804	0.820	0.856	0.499
Organizational loyalty	0.836	0.843	0.884	0.604

**Table 2:** Outer loadings and variance inflation factor (VIF).

**Item**	**Brand-based equity**	**Brand knowledge**	**Organizational culture**	**Organizational loyalty**	**VIF**
BBE	0.708				2.393
BBE1	0.739				1.284
BBE4	0.794				1.480
BBE5	0.793				2.751
BI2		0.768			1.509
BI3		0.852			1.858
BI4		0.883			1.710
OL1				0.850	4.871
OL2				0.766	1.779
OL4				0.679	1.395
OL5				0.798	1.830
OL6				0.784	4.215
RI1			0.786		1.900
RI2			0.739		1.831
RI3			0.619		1.465
T1			0.704		3.019
T2			0.702		4.018
T3			0.678		2.838

The second measure of validity is related to discriminant validity, and for this purpose, it has been assessed through two criteria, namely, Fornell and Larcker (1981) criteria and heterotrait-monotrait (HTMT) ratios (Hair et al., 2017) as depicted from Tables 3, 4. Both criteria were met comfortably as the square root of the AVE of all variables was higher than the correlations in the respective row and column (Hair et al., 2011).

**Table 3:** Discriminant validity (Fornell-Larker criteria).

**Construct**	**Brand-based equity**	**Brand knowledge**	**Organizational culture**	**Organizational loyalty**
Brand-based equity	*0.759*			
Brand knowledge	0.529	*0.836*		
Organizational culture	0.635	0.344	*0.707*	
Organizational loyalty	0.536	0.415	0.357	*0.777*

*Square root of AVE of the respective construct is reported in diagonal values*.

**Table 4:** Discriminant validity [heterotrait-monotrait (HTMT) ratio].

**Construct**	**Brand-based equity**	**Brand knowledge**	**Organizational culture**	**Organizational loyalty**
Brand-based equity	-			
Brand knowledge	0.636	-		
Organizational culture	0.722	0.405	-	
Organizational loyalty	0.644	0.495	0.416	-

While assessing the discriminant validity through HTMT, both liberal and conservative recommendations were followed. From the perspective of liberal criteria, the value of HTMT ratios in Table 4 must be <0.90 while conservative criteria insist that it should be <0.85. Hence, both liberal and conservative criteria have been met (Table 4 to assess discriminant validity through HTMT ratio).

Moreover, model fitness was assessed based on the coefficient of determination (*R*^2^) and effect size (*F*^2^). Model fitness has been found on the bases of these criteria. First, the value of *R*^2^, in this case, is 0.568, indicating that both predictors (brand knowledge and organizational loyalty) along with mediating variable (organizational culture) are explaining 57% variation in the outcome variable (EBBE). Similarly, both predictors (brand knowledge and organizational loyalty) were explaining a 17% variation in mediating variable (organizational culture; Table 5 and Figure 2; Hair et al., 2017). While noting effect size, the value of *F*^2^ has been observed good and depicts the good-quality criteria. Moreover, this study has also tested the model's predictive relevance as recommended by Geisser (1975). The value of *Q*^2^ was assessed, and it has been found greater than zero, depicting a good level of predictive relevance.

**Table 5:** *F*^2^ and adjusted-*R*^2^.

**Construct**	***F*** **-square**	
	**Brand-based equity**	**Organizational culture**
Brand knowledge	0.127	0.056
Organizational culture	**0.385**	**-**
Organizational loyalty	0.127	0.067
**Construct**	***R*** **-square**	
	***R*** **square**	***R*** **square adjusted**
Brand-based equity	0.568	0.563
Organizational culture	0.174	0.168

*Bold values shows significance between the variables*.

**Figure 3:**
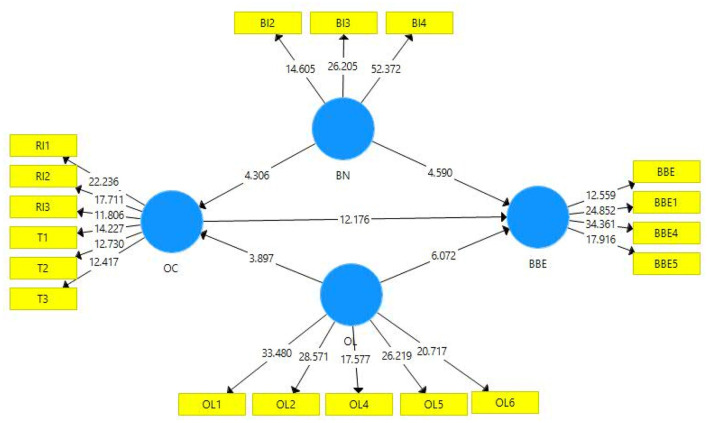
Path significance.

In case of hypotheses testing, it has been performed based on the *t*- and *p*-statistics for each path (**Table 7**). This study anticipated a total of four hypotheses, out of which two were formulated to assess the direct impact while the remaining two were related to the mediation analysis based on mediating variable (organizational culture). The first hypothesis of this study which is related to the relationship between brand knowledge and EBBE has been found statistically significant. In this regard, the value of beta (coefficient) indicated that one unit change in brand knowledge will bring 0.267 unit change in the EBBE (values of *t* and *p*, in this case, were satisfactory, **Table 7**). This state of affairs indicates that brand knowledge has the potency to influence the EBBE positively. Similarly, the second hypothesis of this study is based on the relationship between organizational loyalty and EBBE. This path has been found significant as depicted by *p*- and *t*-statistics in this path (Tables 6, 7 and Figure 3). Thus, it has been found that organizational loyalty influences EBBE positively. Moreover, the coefficient for this path depicts that one unit change in organizational loyalty will bring 0.260 unit change in EBBE. An interesting outcome of this study indicates that both the predictors are sharing almost the same effect in predicting the EBBE. The premise of SET given by Blau (1964) supports these statistically tested hypotheses. Similarly, indirect effects related to mediation were tested based on the variance account for (VAF) approach. For this purpose, the indirect effect was divided through total effect, and the obtained value was multiplied by 100 to calculate the percentage. In case of H3 where it was supposed that organizational culture mediates the relationship between brand knowledge and EBBE, the statistical results indicate that organizational culture is partially mediating this path, which provides sufficient evidence to accept H3. Similarly, H4 was also tested through the VAF approach, and it has been proved that organizational culture mediates the relationship between organizational loyalty and EBBE. These findings are supported through the premise of SET proposed by Blau (1964). These findings are supported through the previous studies (Sekiguchi, 2007; Erkmen, 2018), and it can be safely drawn that employee brand based in firms can be developed through the promotion of knowledge and loyalty within organizational circuits.

**Table 6:** Path estimation.

**Path**	**Coefficient**	***t***	***p***
**Direct paths**			
Brand knowledge → Brand-based equity	0.267	4.590	0.000
Brand knowledge → Organizational culture	0.237	4.306	0.000
Organizational culture → Brand-based equity	0.452	12.176	0.000
Organizational loyalty → Brand-based equity	0.266	6.072	0.000
Organizational loyalty → Organizational culture	0.260	3.897	0.000
**Indirect path**			
Brand knowledge → Organizational culture → Brand-based equity	0.107	4.215	0.000
Organizational loyalty → Organizational culture → Brand-based equity	0.117	3.746	0.000
**Total path**			
Brand knowledge → Brand-based equity	0.374	6.048	0.000
Organizational loyalty → Brand-based equity	0.384	7.680	0.000

**Table 7:** Hypotheses testing.

**Hypotheses**		**Beta**	***t***	***P***	**Status**
H1	Brand knowledge → Brand-based equity	0.267	4.590	0.000	Supported
H2	Organizational loyalty → Brand-based equity	0.266	6.072	0.000	Supported
**Mediation analysis**		**Indirect effect**	**Total effect**	**VAF**	**Status**
H3	Brand knowledge → Organizational culture → Brand-based equity	0.107	0.374	29%	Supported
H4	Organizational loyalty → Organizational culture → Brand-based equity	0.117	0.384	30%	Supported

## Discussion

A lot of research in the past has focused on different aspects of consumer-based brand equity, but very less attention has been given to real antecedents of EBBE. Consumer-based brand equity is becoming more of a concern, but the role of workers is also becoming more prominent (Christodoulides and de Chernatony, 2010). Employees' capacity to execute client expectations is the foundation for building a strong brand and delivering perceived service excellence (King et al., 2013). As the focus of attention changes more and more to employees, experts argue that studying brand equity from the standpoint of employees, dubbed EBBE, is vital (Gounaris, 2006; Mo et al., 2021). Enhancing EBBE helps organizations recruit competent individuals, and workers' skills and experience provide them with a competitive edge. Professionals' identification with enterprises, in contrast, may add to client satisfaction since they connect directly with consumers or customers (Poulis and Wisker, 2016). This research has focused on evaluating the impact of antecedents of equity on EBBE in the hospitality sector.

Brands are made up of a combination of practical and emotional characteristics. To produce a successful commitment to brand image, a brand combines practical and emotional values. As a result, the success of a brand is defined by how effectively it keeps its promises. Providing core competencies in the hotel industry, in contrast, is more challenging. For starters, due to their intangible nature, hospitality services are frequent performances or experiences that are difficult to measure. As a result, articulating the benefits of a brand to customers is more difficult (Erkmen, 2018; Dinçer et al., 2020). Additionally, since consumption and production are intricately intertwined, service providers are commonly misidentified as the service they supply (Erkmen, 2018). Finally, uniformity in hospitality services is difficult to attain because service delivery is dependent on personnel performance. This research evaluated the impact of brand knowledge which is an antecedent of brand equity on EBBE and found a significant relationship between both. This strengthened the previous understanding of brand knowledge in consumer-based brand equity.

It proved that if more attention is given to the brand knowledge of employees through training, then it could result in better developing EBBE. Even though the term “brand knowledge” refers to customers, the idea is also applicable to employees of hospitality services because brand awareness is the cornerstone for building brand equity. Similarly, employees who are familiar with the brand are more likely to grasp their responsibilities and execute the brand promise (Mangold and Miles, 2007; Erkmen, 2018). As a result, brand expertise combined with precise comprehension aids employees in overcoming ambiguity and committing to the brand (de Chernatony and Segal-Horn, 2001; Kumar and Kaushik, 2020; Liu et al., 2020; Osei-Frimpong et al., 2020). Another aspect of EBBE was also studied in this research to assess whether organizational loyalty leads to EBBE or not. The results were significant in identifying that there was a strong positive direct relationship between organizational loyalty and EBBE.

This kind of relationship was not previously studied; hence, it could give some novel insights about hospitality services across the globe for shaping effective EBBE. Few researchers described that loyalty is the most essential element of the ideal company culture and that a loyal employee stays a valuable workforce (Echchakoui, 2015). When companies focus on linking human resources with the company through internal branding, employee loyalty to the brand increases (Du Preez et al., 2017). Therefore, the outcomes of this research are valuable for the marketing practices of internal management in organizations. The last hypotheses were about the evaluation of the mediating role of organizational culture between brand knowledge, organizational loyalty, and EBBE. As supposed, the mediating link of organizational culture also proved to be significant between these antecedents and EBBE.

It suggested that if there is a direct relationship among antecedents with EBBE, then that relationship could be more strengthened by providing an organizational culture to the employees for developing brand equity among them. Every firm has its unique “environment,” which means that understanding organizational culture is crucial for any company seeking a competitive edge through product innovation (Xie et al., 2019). As a result, companies with strong cultural support are better equipped to produce significant advantages in terms of work performance, perspective, moral behavior, ethics, and work happiness, putting themselves in a better position for the long-term business success and competitiveness (Wahyuningsih et al., 2019). A few researchers (Alsheikh et al., 2018; Saleem and Ilkhanizadeh, 2021) have identified the mediating role of organizational culture in different perspectives, but no one has evaluated the mediating role of organizational culture between brand knowledge, organizational loyalty, and EBBE before, so this research would be a novel addition in the field.

### Theoretical and Practical Contribution

This study tends to contribute to the theoretical side of literature from many perspectives: First, this study has tested the EBBE from the perspective of frontline employees of the hospitality sector which is a unique contribution. Second, this study anticipated a mediating mechanism in the shape of organizational culture between the relationship between organizational loyalty and brand knowledge while predicting EBBE. This is another contribution of this study. Similarly, the author tested organizational culture from the perspective of trust and respect for individuals, which is also the contribution of the study. From the practical point of view, this study contends that organizations should try to promote positive knowledge about the firms/organizations to promote a culture of trust and respect for others if they want to promote EBBE.

### Limitations and Future Directions

This study has some potential limitations, such as related to the data collection and the operationalization of study variables. First, this study has only anticipated two dimensions of organizational culture, namely, trust and respect for individuals. Adding other dimensions of organizational culture can provide in-depth insights regarding EBBE. Second, the author has collected data from the hospitality sector, where the frontline employees usually are over-occupied, and thus, collecting data from other sectors can provide more detailed information. Some other study constructs can also be added to the model, such as justice, and its different types, such as procedural, distribute, and interactional justice (De Cuyper et al., 2009; Guest and Clinton, 2017). Other mediating mechanisms can also be tested, such as job satisfaction. Similarly, the moderating variables can also be tested in future studies, such as national culture (power distance and collectivism). Similarly, EBBE can be tested in dimensions in future studies, such as brand allegiance and brand endorsement (Millward and Brewerton, 2000).

## Conclusion

The empirical findings of this study indicate that brand-based equity is influenced by brand knowledge and organizational loyalty. Both organizational loyalty and brand image have almost equal impacts on EBBE. Thus, it can be safely concluded that EBBE is positively influenced at the workplace when employees have a sense of loyalty and knowledge related to the procedures and policies of the firm. Additionally, the impact of organizational culture on EBBE is also positive. Thus, positive organizational culture at the workplace promotes EBBE (Shore et al., 2001, 2006). Furthermore, brand knowledge can also promote a positive culture at the workplace through promoting trust and respect for individuals at the workplace. The same pattern of results indicates that organizational loyalty can also promote a positive culture at the workplace within the organization which further promotes EBBE.

## Data Availability Statement

The original contributions presented in the study are included in the article/supplementary material, further inquiries can be directed to the corresponding author/s.

## Ethics Statement

The studies involving human participants were reviewed and approved by the Shandong Academy of Art, China. The patients/participants provided their written informed consent to participate in this study. The study was conducted in accordance with the Declaration of Helsinki.

## Author Contributions

XL conceived, designed the concept, collected the data, wrote the manuscript, and read and agreed to the published version of the manuscript.

## Conflict of Interest

The author declares that the research was conducted in the absence of any commercial or financial relationships that could be construed as a potential conflict of interest.

## Publisher's Note

All claims expressed in this article are solely those of the authors and do not necessarily represent those of their affiliated organizations, or those of the publisher, the editors and the reviewers. Any product that may be evaluated in this article, or claim that may be made by its manufacturer, is not guaranteed or endorsed by the publisher.

## Appendix

**Table:** Appendix A - Scale and sources

**Scale**	**Items**	**Source**
Organizational loyalty	6	➢ Avoids extra duties at workplace (Negative question) ➢ Does not work beyond what is required (Negative question) ➢ Volunteers for overwork when needed ➢ Is guided by high professional standards ➢ Maintains confidentiality of information ➢ Reports wrongdoing by others
Trust (organizational culture)	3	➢ Most people in my organization can be relied upon to keep their promises ➢ I believe that my colleagues are well-intentioned individuals ➢ I believe that my boss will treat me fairly while appraising my performance
Respect for the individual (organizational culture)	3	➢ My boss trusts me to deliver on his/her expectations ➢ My supervisor believes that good ideas and solutions to problems can come from any member of the group ➢ My organization makes the best possible use of my intellectual capacity
EBBE	5	➢ I am aware that our brand significantly contributes to the overall success of our company. ➢ I am convinced that our brand allows us to achieve a higher price for our products. ➢ I believe that our customers buy higher quantities due to our brand. ➢ I believe that our brand accounts considerably for the loyalty of our customers. ➢ I am convinced that our customers recommend our brand to others
Brand knowledge (internal branding)	4	➢ The (brand) values are reinforced through internal communications ➢ Training is provided to help employees use these values ➢ The skill set necessary to deliver these values is considered in staffing decisions ➢ Annual performance reviews include metrics on delivering the values
